# Artificial intelligence anxiety and its association with willingness to receive AI-related training among maternal and child health nurses: a multicenter network analysis

**DOI:** 10.3389/fpubh.2026.1889617

**Published:** 2026-08-21

**Authors:** Cheng Huang, Qin Zeng, Jun Zhu, Yuji Wang, Xi Huang

**Affiliations:** 1 National Office for Maternal and Child Health Surveillance of China, West China Second University Hospital, Sichuan University, Chengdu, Sichuan, China; 2 Key Laboratory of Birth Defects and Related Diseases of Women and Children, Sichuan University, Ministry of Education, Chengdu, Sichuan, China; 3 Department of Pediatric Gastroenterology Nursing, West China Second University Hospital, Sichuan University, Chengdu, China; 4 Department of Neonatology Nursing, West China Second University Hospital, Sichuan University, Chengdu, China

**Keywords:** anxiety, artificial intelligence, maternal and child health nursing, network analysis, nursing management, staff development

## Abstract

**Background:**

Rapid integration of artificial intelligence (AI) into maternal and child healthcare introduces unique psychological challenges for nursing staff. This study aimed to explore the network structure of AI anxiety among Chinese maternal and child health nurses and examine its associations with willingness to receive AI-related training, thereby generating preliminary implications for organizational support strategies.

**Methods:**

A multicenter cross-sectional survey was conducted between May and August 2025, enrolling 848 nurses from 46 maternal and child healthcare institutions across 26 Chinese provinces. AI anxiety was assessed using the Artificial Intelligence Anxiety Scale (AIAS), and willingness to receive AI-related training was assessed using a single 10-point item. Network analysis using the Least Absolute Shrinkage and Selection Operator (LASSO) and the Extended Bayesian Information Criterion (EBIC) was performed to estimate statistically central nodes, cross-dimensional bridge connectivity, and node predictability.

**Results:**

The highest strength centrality values were observed for a humanoid-AI-fear item and a learning-anxiety item. AI misuse concerns and job replacement fears showed the highest bridge-strength values, indicating relatively strong cross-dimensional connectivity among the anxiety dimensions. Notably, learning-anxiety items showed the strongest positive associations with willingness to receive AI-related training, with edge weights ranging from 0.22 to 0.24. The estimated strength and bridge-strength rankings showed acceptable stability, with correlation-stability coefficients of 0.594 and 0.750, respectively, while node predictability ranged from 69.3 to 92.1%.

**Conclusion:**

AI anxiety among maternal and child health nurses showed a multifaceted network structure in which learning-related stress and fear of anthropomorphic AI were statistically prominent. These data generate preliminary hypotheses about organizational support but do not identify effective intervention targets. Because willingness to receive AI-related training was assessed using a single global item, the study does not identify nurses' multidimensional educational needs, preferred content or delivery modalities, competency priorities, or perceived barriers. Literature-informed options such as phased or simulation-based training should therefore undergo needs assessment and longitudinal or interventional evaluation before implementation.

## 1 Introduction

In recent years, the rapid development of artificial intelligence (AI) technology has revolutionized medical practice, demonstrating significant potential in the field of maternal and child health. Howell et al. (1) described the evolution of healthcare AI from task-specific classification and prediction toward foundation models and generative AI, highlighting its expanding clinical potential alongside emerging risks. In mainland China, however, maternal and child health institutions are gradually adopting AI technologies to address rising service demands and complex clinical scenarios. Maternal and child health nurses play a pivotal role in the healthcare system; their service quality is significantly associated with the health outcomes of mothers and children (2). These nurses perform high-intensity, high-risk clinical tasks, such as labor monitoring and neonatal intensive care, while also providing sustained emotional and humanistic support to mothers, children, and families (3, 4). Consequently, AI-related changes may affect both their technical work and the relational core of their professional role.

Within this context, adjustments to fertility policies and intensifying population aging have increased the demand for maternal and child health services, while nurse shortages and uneven resource allocation have further complicated service delivery (5). The International Council of Nurses reported that 40–80% of nurses globally experience psychological distress and that at least 20% intend to leave their jobs. The organization therefore described the nursing shortage as a global health emergency (6).

Against this backdrop, while the integration of AI technology bolsters operational efficiency, it simultaneously introduces novel psychological stressors for maternal and child health nurses. As a group providing direct services to mothers and children, nurses must rapidly adapt to new technologies; however, intense work pressure and complex emotional demands may lead to the development of AI anxiety. This anxiety comprises four dimensions (7, 8): (1) Learning Anxiety, referring to anxiety about mastering and using AI technologies; (2) Job Replacement Anxiety, referring to concerns that AI may replace human labor or erode professional competence; (3) Sociotechnical Blindness, referring to concerns about AI misuse, loss of control, or bias; and (4) AI Configuration Anxiety, referring to fear or discomfort related to anthropomorphic AI, such as humanoid robots. AI misuse, loss of control, or bias; and (4) AI Configuration Anxiety, referring to fear or discomfort related to anthropomorphic AI, such as humanoid robots.

Othman et al. (7) found that 28.5% of nurses exhibited severe anxiety before engaging with AI technologies, with learning difficulties being a primary stressor, particularly in the absence of training. Amin et al. (9) further noted that some nurses fear that AI, despite improving efficiency, may reduce interpersonal interactions and exacerbate job insecurity.

In China's maternal and child health sector, the nascent stage of AI adoption and inadequate training amplify technological unfamiliarity, while the emotional support demands of the role make nurses particularly sensitive to role-related conflicts introduced by AI (10). Recent research in Chinese maternal and child health settings underscores the dynamic relationship between nurses' AI self-efficacy and usage demands, revealing that training deficiencies significantly hinder technology integration (11). Furthermore, AI literacy has been identified as a critical precursor that shapes usage intention through self-efficacy and attitude pathways, with notable regional disparities across Beijing, Sichuan, and Yunnan (12). AI anxiety may shape nurses' responses to technology adoption; however, its relationships with care quality, job satisfaction, professional wellbeing, and retention require direct empirical examination.

An updated targeted search of PubMed, Web of Science, and Scopus identified several nursing studies with similar objectives, which mainly used descriptive comparisons, correlation analyses, and regression- or mediation-based models to examine overall or dimensional AI anxiety scores and their associations with readiness, attitudes, and related factors (7, 13–15). Although informative, these variable-centered approaches do not reveal item-level conditional dependencies, central nodes, or bridge connections across anxiety dimensions (16, 17). Additionally, AI anxiety may be associated with nurses' willingness to receive AI-related training, which provides a limited indicator of overall training demand but does not represent a comprehensive assessment of multidimensional training needs. However, no study identified in this search has examined the item-level network structure of AI anxiety or its association with willingness to receive AI-related training specifically among Chinese maternal and child healthcare nurses.

Network analysis, as an emerging method, can characterize conditional association patterns and identify statistically central or bridging components within the AI anxiety network by constructing a network model of psychological items (16, 17). This methodology has previously been used to characterize the architecture of AI self-efficacy among nurses, identifying anthropomorphic interaction as a statistically central feature within the estimated network (11).

This study, based on the Artificial Intelligence Anxiety Scale (AIAS), adopts network analysis to systematically explore the structure of AI anxiety among Chinese maternal and child healthcare nurses and its relationship with willingness to receive AI-related training. The research focuses on the relative network positions and connectivity patterns of learning anxiety and humanoid-AI fear. The study aims to answer the following questions: (1) What are the structural characteristics of the AI anxiety network? (2) Which anxiety items occupy central positions in the network? (3) How is AI anxiety associated with willingness to receive AI-related training, and how might these findings generate preliminary implications for organizational support strategies?

The theoretical significance of this study lies in revealing the complex associations of AI anxiety through network analysis, providing a new perspective for nursing psychology. The practical significance of this study is that it generates preliminary hypotheses for the prospective development and evaluation of training and organizational support strategies for maternal and child healthcare nurses. However, the single-item measure of training willingness does not provide a comprehensive assessment of nurses' specific training needs or preferences. Moreover, because nursing performance, care quality, job satisfaction, professional wellbeing, and retention were not assessed, the findings should not be interpreted as demonstrating improvements in these outcomes; any potential effects on these outcomes require further empirical evaluation.

## 2 Subjects and methods

### 2.1 Study subjects

This study adopted a cross-sectional survey design and used convenience sampling to recruit nurses practicing in maternal and child healthcare institutions in mainland China from May 1, 2025, to August 31, 2025. The inclusion criteria were as follows: being a registered nurse currently engaged in clinical practice; having at least 1 year of clinical teaching experience; and providing voluntary consent to participate.

The exclusion criteria were as follows: nurses who had taken continuous leave for more than 6 months during the preceding year; nurses who were temporarily not engaged in routine clinical nursing and clinical teaching duties during the data-collection period because of full-time off-site training, full-time academic study or research assignments, external continuing education, probationary employment without independent clinical or teaching responsibilities, or post-retirement appointments without regular frontline clinical or teaching duties; nurses who had previously participated in similar questionnaire surveys. Questionnaires completed in less than 3 min or more than 30 min were considered invalid and excluded during data cleaning.

These role-based exclusions were applied to ensure that participants had current and sustained exposure to routine clinical nursing and clinical teaching environments during the study period. Educational attainment, previous research experience, and prior AI-related training were not exclusion criteria; nurses with postgraduate degrees, research experience, or previous AI training remained eligible if they were actively engaged in routine clinical practice and met the other inclusion criteria. Specifically, this study focused on clinical nursing instructors to ensure that the data reflected the perspectives of those responsible for both frontline care and the professional development of junior staff. This focus may partly explain the relatively high proportion of participants recruited from teaching and tertiary hospitals.

### 2.2 Research instruments

#### 2.2.1 General information questionnaire

Designed by the researchers, this questionnaire included items such as self-reported sex (male or female), age, ethnicity, marital status, educational level, professional title, years of work experience, department, whether the participant is a clinical instructor, hospital type, hospital level, and whether the hospital is a teaching institution. The questionnaire assessed sex rather than gender identity.

#### 2.2.2 Artificial intelligence anxiety scale

The AIAS, developed by Wang and Wang (8), was used to assess AI anxiety among nurses. Although the original study initially examined the scale in relation to motivated learning behavior, the AIAS was developed as a general measure of anxiety elicited by AI technologies rather than as an education-specific instrument. Its four dimensions, Learning Anxiety, Job Replacement Anxiety, Sociotechnical Blindness, and AI Configuration Anxiety, are conceptually relevant to clinical nursing because they reflect concerns about acquiring AI-related competencies, occupational displacement, technology misuse or loss of control, and discomfort with anthropomorphic AI systems (8). The scale has subsequently been applied in nursing populations, supporting its relevance beyond its original educational application (13–15).

The AIAS consists of 21 items, divided into four dimensions: learning anxiety (eight items, anxiety about learning AI technologies), Job Replacement Anxiety (six items, concerns that AI may replace their jobs), Sociotechnical Blindness (four items, worries about potential issues with AI technologies), and AI Configuration Anxiety (three items, fear of humanoid AI). The scale uses a 7-point Likert response format, ranging from 1 (“strongly disagree”) to 7 (“strongly agree”), with total scores ranging from 21 to 147. Higher scores indicate greater AI anxiety. In this study, Cronbach' s α was 0.975 for the total AIAS and 0.979, 0.955, 0.944, and 0.978 for the Learning Anxiety, Job Replacement Anxiety, Sociotechnical Blindness, and AI Configuration Anxiety dimensions, respectively, indicating excellent internal consistency.

#### 2.2.3 Assessment of willingness to receive AI-related training

To assess maternal and child healthcare nurses' willingness to receive AI-related training, a separate supplementary item was administered alongside the AIAS. Participants self-rated their response to the question, “Considering various factors, what is your level of willingness to receive training on AI-related knowledge?” using a 10-point Likert scale, with scores ranging from 1 (“completely unwilling”) to 10 (“very willing”). The score was treated as an indicator of participants' overall willingness to receive AI-related training rather than as a comprehensive multidimensional assessment of training needs.

The single-item format was selected because the target variable represented a narrowly defined global judgment and because a brief measure could reduce respondent burden in a survey that already included the 21-item AIAS. Previous methodological studies have shown that well-defined single-item measures can demonstrate adequate criterion and predictive validity in specific contexts, particularly when the target construct is concrete and narrowly defined (18–20). Nevertheless, this single global item cannot capture the multidimensional nature of training needs, including preferred educational content, learning modalities, required technical competencies, ethical and safety concerns, or perceived barriers to training participation. Accordingly, analyses involving this item were interpreted as reflecting general willingness to receive AI-related training rather than comprehensive or domain-specific training needs.

### 2.3 Survey methods and quality control

#### 2.3.1 Sample size calculation

This study used the standard sample-size formula for cross-sectional studies (21, 22):


$$\begin{array}{l} n={\left[\frac{Z_{\left(1-\alpha /2\right)}}{\delta }\right]}^{2}\times p\times \left(1-p\right) \end{array}$$

Where: Z_(1−α/2)_ is the critical value of the standard normal distribution. At α = 0.05, Z_(1−α/2)_ was set at 1.96. To maximize the required sample size, *p* was set at 0.50, and the allowable error, δ, was set at 0.05 (5%). The resulting minimum sample size was 385 participants. After allowing for a 20% rate of invalid or incomplete questionnaires, the target sample size was 462 participants.

#### 2.3.2 Pilot study

To evaluate and enhance the contextual applicability of the Chinese version of the AIAS, the scale was translated and culturally adapted using the Brislin back-translation model. First, two nursing experts with medical backgrounds and proficiency in both Chinese and English performed independent forward translations. Subsequently, an English language expert who was blinded to the original scale conducted a back-translation into English. The research team then compared the back-translated version with the original scale to ensure semantic equivalence.

Following the translation process, six senior experts with experience in AI, psychology, and sociology were consulted to evaluate the content validity of the scale items. The expert panel focused on assessing the accuracy of the item descriptions within the clinical context of maternal and child healthcare. Based on their recommendations, localized modifications were made to the Chinese terminology for specific terms, such as “humanoid AI”. The results showed an Item Content Validity Index (I-CVI) ranging from 0.83 to 1.00, and an overall Scale Content Validity Index (S-CVI) of 0.992, indicating excellent content validity. Together, the translation, expert review, and content-validity assessment supported the linguistic clarity and content relevance of the AIAS for maternal and child healthcare nurses in the local clinical context.

Prior to the formal survey, a pilot study was conducted with a sample size set at 10% to 30% of the total sample (23). Based on the calculated formal survey sample size of 462 participants, the pilot study sample size ranged from 46 to 139 individuals. Using convenience sampling, 50 nurses from a tertiary maternal and child healthcare institution in southwestern China were selected for the pilot test to assess the clarity and comprehensibility of the questionnaire.

Based on the pre-test feedback, the participating nurses reported that the scale language was concise, easy to understand, and aligned with their daily work context. Consequently, necessary modifications and adjustments were made to the questionnaire content to ensure that all items were accurate and easily comprehensible. The pilot test results showed a Cronbach' s α internal consistency coefficient of 0.977 for the AIAS, with subscale α values of Learning (α = 0.980), Job Replacement (α = 0.976), Sociotechnical Blindness (α = 0.969), and AI Configuration (α = 0.991), indicating excellent internal consistency.

#### 2.3.3 Formal survey

The formal survey was conducted by distributing electronic questionnaires via the Wenjuanxing platform (https://www.wjx.cn/), a professional and efficient online tool for questionnaire design and data collection widely used in mainland China for its convenience, security, reliability, and anonymity. A QR code for the questionnaire was generated through the platform and distributed via email and social media (e.g., WeChat) to head nurses at various maternal and child healthcare institutions. The head nurses then forwarded the questionnaire to potential participants within their departments to ensure effective coverage and response collection.

#### 2.3.4 Quality control

The study used encrypted electronic questionnaires (via the Wenjuanxing platform) to collect data, and multiple quality control measures were implemented to ensure data quality. Participants were required to first read the electronic informed consent form and confirm their agreement before proceeding with the questionnaire. The questionnaire included standardized instructions and a mandatory response function, with a page-by-page design to ensure complete responses for each section. All submissions were anonymous, and each IP address was limited to submitting once. The research team monitored the completion progress and data quality in real time through the platform's backend. These measures were used to enhance data integrity and protect participant privacy.

#### 2.3.5 Data export and analysis

After data collection was completed, all questionnaire data were exported from the Wenjuanxing platform in Excel format. The exported data included the complete responses of the respondents, the specific response time for each question, and the total completion time. Following export, the researchers cleaned and organized the data, removing invalid responses and obviously anomalous data. The survey covered 46 maternal and child healthcare institutions across 26 provinces in mainland China.

### 2.4 Statistical analysis

This study utilized *R* software (version 4.4.3) for data analysis, with network analysis primarily conducted using the qgraph and bootnet packages. First, descriptive statistics were generated for all variables: continuous variables were expressed as mean±standard deviation, while categorical variables were presented as frequencies and percentages. To assess the degree of variation for each item in the AIAS, standard deviation was used as an indicator of information content. Additionally, item redundancy was evaluated by examining inter-item correlations to ensure that there was no excessive overlap between scale items (17). Because AI anxiety and willingness to receive AI-related training were assessed through self-report by the same participants in a single survey at one time point, the observed associations were considered potentially susceptible to common method variance and were interpreted cautiously.

#### 2.4.1 Estimation and validation of the AIAS network structure

Network analysis is a data-driven approach for estimating conditional associations among variables within a complex system (16). In this study, network analysis was used to examine the conditional association patterns among the 21 AIAS items. The analysis was conducted using the qgraph package in *R* software to construct and visualize the network structure, transforming the partial correlation matrix into a network graph displaying non-zero regularized partial correlations. The AI anxiety network structure was built using LASSO-regularized partial correlation analysis, which reduces noise through regularization to produce a sparser network, emphasizing key associations between anxiety items (16). Model selection was optimized using the Extended Bayesian Information Criterion to balance model fit and parsimony (17).

Network visualization was based on the Fruchterman–Reingold spring layout, in which more strongly connected nodes tend to be positioned closer together (24). In the network constructed for this study, nodes represent the 21 anxiety items of the AIAS (e.g., “Learning to understand all of the special functions associated with AI technologies makes me anxious”), while edges represent the partial correlation coefficients between two items after controlling for all other items. The thickness of the edges reflects the strength of the association: stronger associations are depicted by thicker edges, while weaker associations are shown with thinner edges (17). Green edges indicate positive correlations, and red edges indicate negative correlations. Through this approach, the network graph provides a clear and intuitive visualization of the complex structure of nurses' AI-related anxiety.

The primary network metrics interpreted in this study were strength, bridge strength, and predictability. Expected influence and bridge expected influence, which retain the direction of the estimated edge weights, were also calculated as supplementary signed-connectivity indices; however, the main interpretation focused on strength and bridge strength. Node strength is the sum of the absolute weights of all edges connected to a given node (25), quantifying the node's aggregate absolute connectivity within the estimated network. A higher strength value indicates stronger overall conditional associations with other nodes but does not establish greater causal influence. For example, it indicates the extent to which “I am afraid that AI technologies may replace humans” is statistically connected with other anxiety items in the estimated network. Bridge strength is the sum of the absolute edge weights connecting a node to nodes in other dimensions, reflecting its cross-dimensional connectivity (26). Bridge strength describes statistical connectivity across pre-defined dimensions and should not be interpreted as evidence that a node causally transmits or amplifies anxiety across dimensions. For instance, it assesses whether a learning-anxiety item is associated with job-replacement-anxiety items. Predictability reflects the extent to which an anxiety item can be predicted by other items (27). For example, it evaluates the extent to which the variance in “I am afraid that widespread use of humanoid robots will take jobs away from people” is statistically accounted for by neighboring anxiety items, such as “Learning to use AI technologies makes me anxious.” In the present study, strength, bridge strength, and predictability were interpreted as descriptive indices of relative network position and statistical connectivity. Because the network was undirected and estimated from cross-sectional data, higher values were not interpreted as evidence of temporal precedence, causal influence, or intervention effectiveness (17, 28).

To assess the reliability of the network-analysis results, edge-weight accuracy and centrality stability were evaluated (17, 28). Edge-weight accuracy was assessed using 95% non-parametric confidence intervals based on 1,000 bootstrap samples. Centrality stability was evaluated for both node strength and bridge strength using correlation-stability coefficients. Differences in edge weights, node strengths, and bridge strengths were examined using bootstrap confidence intervals.

#### 2.4.2 Analysis of the association between AI anxiety and willingness to receive AI-related training

To identify which AIAS items were most closely associated with willingness to receive AI-related training, the training-willingness item was integrated into the AI anxiety network. A LASSO-regularized partial correlation analysis was used to estimate the association between this node and each anxiety item. The resulting edge weights were visualized to identify the AI anxiety items most closely associated with the global training-willingness rating among maternal and child health nurses. This analysis did not assess specific training-content or delivery preferences.

## 3 Results

### 3.1 Sample characteristics and general information

A total of 985 questionnaires were returned. Of these, 137 were excluded: 35 were completed by participants without the required clinical teaching experience, 48 were completed by respondents who were not engaged in routine clinical nursing and teaching duties during the data-collection period, and 54 were completed in less than 3 min. Accordingly, 848 valid questionnaires were included in the analysis, yielding a valid-questionnaire rate of 86.09%.

As shown in Table 1, 97.1% of the participants were female (823/848). The majority were aged 31–40 years (47.2%, 400 individuals), followed by 18–30 years (35.8%, 304 individuals). Han ethnicity accounted for 92.1% (781 individuals), while other ethnic groups comprised 7.9% (67 individuals). Participants who were married or living with a partner accounted for 73.7% of the sample (625 individuals). In terms of education, the majority held a bachelor's degree (69.1%, 586 individuals), followed by junior college or below (22.3%, 189 individuals), and master's degree or above (8.6%, 73 individuals).

**Table 1:** Characteristics of the clinical nursing instructors (*N* = 848).

Characteristics	Frequency, *n*	Percentage, %
Sex		
Male	25	2.9
Female	823	97.1
Age (years)		
18–30	304	35.8
31–40	400	47.2
41–50	126	14.9
51–60	18	2.1
Ethnicity		
Han	781	92.1
Others	67	7.9
Marital status^*^		
Married or partnered	625	73.7
Not married or partnered	223	26.3
Educational level		
Junior college degree or below	189	22.3
Bachelor's degree	586	69.1
Master's degree or above	73	8.6
Working experience (years)		
≤ 5	189	22.3
6–10	219	25.8
11–15	237	27.9
≥16	203	23.9
Professional title		
Junior	401	47.3
Intermediate	383	45.2
Senior	64	7.5
Work department^**^		
Obstetrics and gynecology	216	25.5
Pediatrics	394	46.5
Maternal and child health	238	28.1
Job content		
Direct clinical care	779	91.9
Administration, teaching, research, or other duties	69	8.1
Training experience in artificial intelligence (AI)		
Received specialized training	116	13.7
No specialized training, but has been exposed to related topics or self-studied	362	42.7
No prior exposure to the topic	370	43.6
Hospital type		
General hospital	416	49.1
Specialized hospital	432	50.9
Hospital level^***^		
Tertiary grade A	491	57.9
Tertiary grade B	137	16.2
Secondary grade	220	25.9
Teaching hospital status		
Yes	609	71.8
No	239	28.2

^*^Married or partnered included participants who were married or partnered; not married or partnered included those who were never married, separated, divorced, or widowed.

^**^In this study, maternal and child health nurses were categorized into three major departments based on their service recipients:

**Obstetrics and gynecology:** this department focuses on the care of pregnant women, postpartum women, and women's reproductive health, encompassing prenatal, intrapartum, and postnatal care, as well as the management of gynecological conditions.

**Pediatrics:** This department provides healthcare for children from newborns to adolescents, including disease prevention, vaccination, and monitoring of growth and development.

**Maternal and child health:** this department integrates health promotion, disease prevention, and health education for both women and children.

^***^ Hospital level indicates the hospital's capacity and quality in China. Tertiary grade A: top-tier hospitals with highest-level services and advanced technology. Tertiary grade B: similar to grade A but slightly lower in capacity. Secondary grade: Mid-level facilities serving smaller regions.

Work experience was broadly distributed across the four categories, with 11–15 years being the most common (27.9%, 237 individuals), followed by 6–10 years (25.8%, 219 individuals), 16 years or more (23.9%, 203 individuals), and 5 years or less (22.3%, 189 individuals). Professional titles were mainly junior (47.3%, 401 individuals) and intermediate (45.2%, 383 individuals). The most common work department was pediatrics (46.5%, 394 individuals), followed by maternal and child health (28.1%, 238 individuals) and obstetrics and gynecology (25.5%, 216 individuals). Nurses engaged in frontline clinical care accounted for 91.9% (779 individuals), while those in administrative, teaching, research, or other roles comprised 8.1% (69 individuals).

Regarding AI training experience, 43.6% (370 individuals) had never been exposed to AI-related content, 13.7% (116 individuals) had received specialized training, and 42.7% (362 individuals) had not received specialized training but had self-studied or encountered related topics. Thus, the analytical sample was not restricted to nurses with limited AI-related knowledge or exposure, as 56.4% of the participants had received specialized AI training or had acquired AI-related knowledge through self-directed learning or other forms of exposure.

General hospitals and specialized hospitals accounted for 49.1% (416/848) and 50.9% (432/848) of the sample, respectively. Tertiary Grade A hospitals comprised 57.9% (491 individuals), Tertiary Grade B hospitals 16.2% (137 individuals), and secondary hospitals 25.9% (220 individuals). Additionally, 71.8% (609 individuals) of the nurses worked in teaching hospitals, while 28.2% (239 individuals) worked in non-teaching hospitals.

### 3.2 Measurement tools and core variable analysis

Table 2 presents the means and standard deviations of the 21 AIAS items. No item met the pre-specified criterion for low informativeness, and no item pair met the pre-specified criterion for redundancy; therefore, all 21 items were retained. The mean score for willingness to receive AI-related training was 5.82 ± 1.76.

**Table 2:** Descriptive statistics for the artificial intelligence anxiety scale dimensions and items.

Items	Mean	SD
Learning anxiety	32.02	10.253
L_1. Learning to understand all of the special functions associated with AI technologies makes me anxious	4.11	1.355
L_2. Learning to use AI technologies makes me anxious	4.00	1.380
L_3. Learning to use specific functions of AI technologies makes me anxious	4.00	1.373
L_4. Learning how AI technology works makes me anxious	4.03	1.400
L_5. Learning to interact with AI technologies makes me anxious	3.91	1.380
L_6. Taking a class about the development of AI technologies makes me anxious	3.95	1.392
L_7. Reading an AI technology manual makes me anxious	3.96	1.394
L_8. Being unable to keep up with the advances associated with AI technologies makes me anxious	4.07	1.391
Job replacement anxiety	27.46	7.549
J_1. I am afraid that AI technologies may make us dependent	4.65	1.347
J_2. I am afraid that AI technologies may make us even lazier	4.61	1.365
J_3. I am afraid that AI technologies may replace humans	4.29	1.488
J_4. I am afraid that widespread use of humanoid robots will take jobs away from people	4.63	1.431
J_5. I am afraid that if I begin to use AI technologies, I will become dependent upon them and lose some of my reasoning skills	4.61	1.399
J_6. I am afraid that AI technologies will replace someone's job	4.67	1.418
Sociotechnical blindness	18.61	5.091
S_1. I am afraid that AI technologies may be misused	4.74	1.407
S_2. I am afraid of various problems potentially associated with AI technologies	4.69	1.366
S_3. I am afraid that AI technologies may get out of control and malfunction	4.67	1.393
S_4. I am afraid that AI technologies may lead to robot autonomy	4.51	1.424
AI configuration anxiety	12.28	4.383
C_1. I find humanoid AI technologies (e.g., humanoid robots) scary	4.13	1.493
C_2. I find humanoid AI technologies (e.g., humanoid robots) intimidating	4.10	1.492
C_3. I don't know why, but humanoid AI technologies (e.g., humanoid robots) scare me	4.04	1.517

SD, standard deviation; L, learning anxiety; J, job replacement anxiety; S, sociotechnical blindness; C, AI configuration anxiety.

Terms such as “AI technique/product” or “AI techniques/products” from the original AIAS scale have been standardized as “AI technology” or “AI technologies” throughout this manuscript to ensure terminological consistency and conceptual clarity, without altering the underlying psychological constructs of the items.

### 3.3 AI anxiety network feature analysis

#### 3.3.1 Network structure characteristics

**Figure 1:**
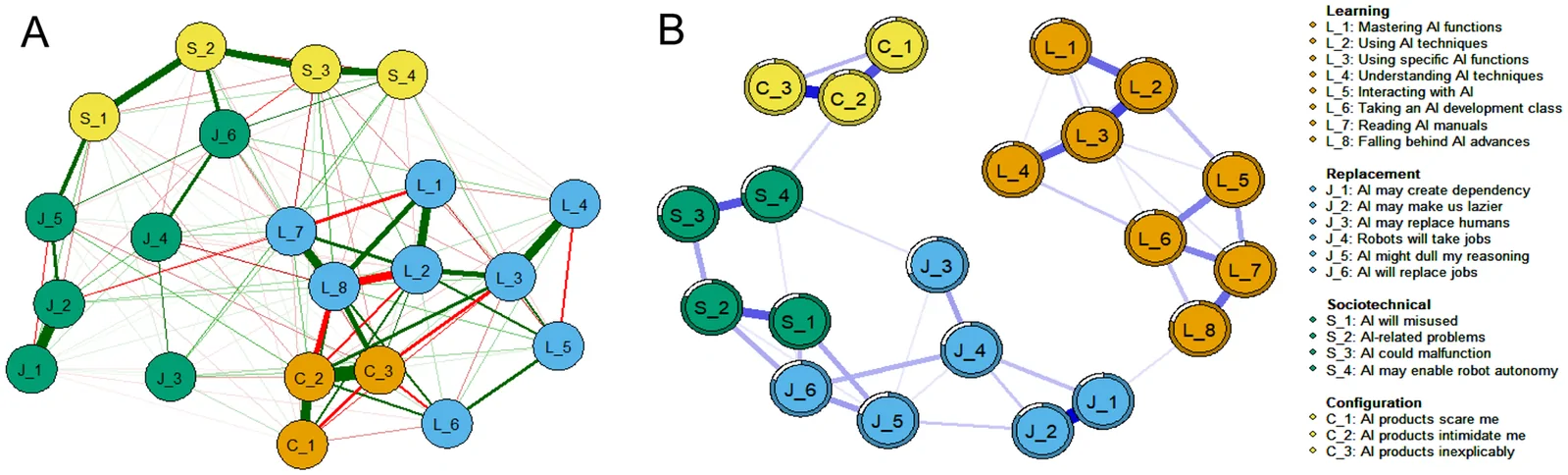
AIAS network structure and node predictability (*N* = 848). **(A)** AI anxiety network structure: this panel illustrates the conditional association structure among the 21 AIAS items. Nodes represent individual anxiety items, with colors corresponding to four dimensions: Learning Anxiety (blue, L1–L8), Job Replacement Anxiety (green, J1–J6), Sociotechnical Blindness (yellow, S1–S4), and AI Configuration Anxiety (orange, C1–C3). Edges represent regularized partial correlation coefficients between pairs of items after conditioning on all other items in the network. Edge thickness reflects the magnitude of the association, while edge color indicates its direction: green denotes positive associations and red denotes negative associations. **(B)** Node predictability: the shaded rings around each node represent predictability, defined as the proportion of variance in each item statistically accounted for by its neighboring nodes in the fitted network (*R*^2^). A more complete ring indicates that a larger proportion of the variance in the corresponding item is statistically accounted for by neighboring nodes. Predictability does not establish causal influence among the items.

Figure 1A illustrates the network structure of the AIAS, comprising 21 nodes, each representing one of the 21 anxiety items. These items span four dimensions: Learning (L_1 to L_8), Job Replacement (J_1 to J_6), Sociotechnical Blindness (S_1 to S_4), and AI Configuration (C_1 to C_3). The network contained 87 non-zero edges, representing 41.43% of all possible edges (87/210), with a mean edge weight of 0.049. Edges were calculated using a graphical partial correlation matrix, displaying non-zero regularized partial correlations. Green edges represent positive correlations, red edges represent negative correlations, and deeper colors and thicker widths indicate stronger associations.

#### 3.3.2 Strength centrality analysis

**Figure 2:**
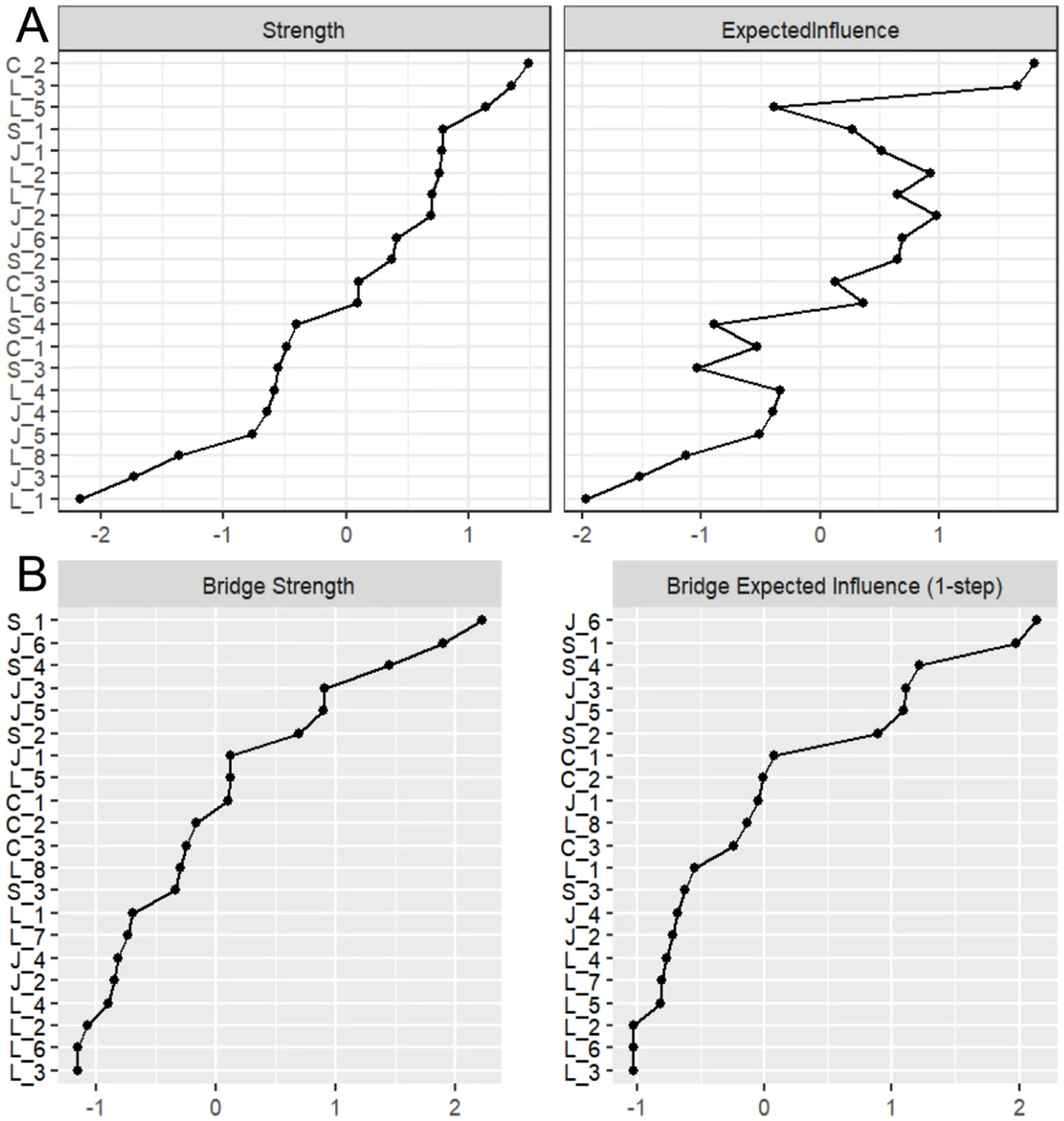
Centrality and bridge centrality indices for the AIAS network. **(A)** Node strength and expected influence**:** this panel presents the standardized centrality scores for each of the 21 AIAS items. Strength represents the sum of the absolute weights of all edges connected to a specific node and reflects its aggregate absolute connectivity within the estimated network. Expected Influence is a signed-connectivity index that accounts for both the magnitude and the direction, positive or negative, of the estimated edge weights. Items with higher standardized values, such as C_2 and L_3, showed relatively greater statistical connectivity within the estimated network. **(B)** Bridge strength and bridge expected influence: this panel presents the cross-dimensional connectivity of nodes across the predefined AI anxiety dimensions. Bridge Strength represents the sum of the absolute weights of edges connecting a node to nodes in other predefined dimensions and reflects its aggregate cross-dimensional connectivity within the estimated network. Bridge Expected Influence (1-step) is a signed-connectivity index that accounts for the magnitude and direction of a node's estimated connections with nodes in other dimensions. Nodes with higher bridge values, such as S_1 and J_6, showed relatively greater cross-dimensional statistical connectivity within the estimated network. These indices describe statistical connectivity within the estimated cross-sectional network and do not establish causal influence, temporal ordering, symptom transmission, clinical importance, or intervention effectiveness.

Figure 2A and Table 3 present the Strength and Expected Influence of the 21 nodes. The results show that C_2 from the AI Configuration Anxiety dimension (“I find humanoid AI technologies (e.g., humanoid robots) intimidating”) exhibits the highest strength centrality (Strength = 1.488), indicating the highest aggregate absolute connectivity among the estimated nodes. This is followed by L_3 from the Learning Anxiety dimension (“Learning to use specific functions of AI technologies makes me anxious,” Strength = 1.350) and L_5 (“Learning to interact with AI technologies makes me anxious,” Strength = 1.142).

**Table 3:** Standardized centrality estimates and predictability of the AIAS network nodes.

Nodes	Variable	Standardized strength (z)	Standardized bridge strength (z)	Predictability (*R*^2^)
L_1	Mastering AI functions	−2.166	−0.689	0.804
L_2	Using AI technologies	0.757	−1.073	0.907
L_3	Using specific AI functions	1.350	−1.147	0.921
L_4	Understanding AI technologies	−0.583	−0.894	0.871
L_5	Interacting with AI	1.142	0.118	0.864
L_6	Taking an AI development class	0.090	−1.147	0.870
L_7	Reading AI manuals	0.700	−0.737	0.872
L_8	Falling behind AI advances	−1.356	−0.291	0.796
J_1	AI may create dependency	0.781	0.118	0.855
J_2	AI may make us lazier	0.688	−0.847	0.852
J_3	AI may replace humans	−1.729	0.907	0.693
J_4	Robots may take jobs	−0.648	−0.811	0.778
J_5	AI may dull my reasoning	−0.759	0.893	0.794
J_6	AI may replace jobs	0.414	1.899	0.805
S_1	AI may be misused	0.794	2.215	0.834
S_2	AI-related problems	0.374	0.695	0.819
S_3	AI may malfunction	−0.550	−0.337	0.739
S_4	AI may enable robot autonomy	−0.407	1.442	0.756
C_1	Humanoid AI scares me	−0.481	0.098	0.858
C_2	Humanoid AI intimidates me	1.488	−0.167	0.917
C_3	Humanoid AI inexplicably scares me	0.100	−0.247	0.896

Variable labels are abbreviated descriptions of the corresponding AIAS items; the full item wording is provided in Table 2. Strength and bridge strength are presented as standardized z scores. Predictability is reported as *R*^2^. Centrality indices describe statistical connectivity within the estimated network and do not establish causal or clinical importance.

#### 3.3.3 Bridge strength analysis

Figure 2B and Table 3 display the results of bridge strength and bridge expected influence analyses. S_1 from the Sociotechnical Blindness dimension (“I am afraid that AI technologies may be misused”) demonstrates the highest bridge strength (Bridge Strength = 2.215), indicating the greatest estimated cross-dimensional connectivity among the included nodes (e.g., Learning Anxiety and Job Replacement Anxiety). This is followed by J_6 from the Job Replacement Anxiety dimension (“I am afraid that AI technologies will replace someone's job,” Bridge Strength = 1.899) and S_4 from the Sociotechnical Blindness dimension (“I am afraid that AI technologies may lead to robot autonomy,” Bridge Strength = 1.442).

#### 3.3.4 Node predictability

Figure 1B and Table 3 illustrate the predictability of the 21 nodes, represented by circles around each node, with values ranging from 69.3% to 92.1%. L_3 from the Learning Anxiety dimension (“Learning to use specific functions of AI technologies makes me anxious”) exhibits the highest predictability (*R*^2^ = 0.921), meaning that 92.1% of its variance was statistically accounted for by its neighboring items under the fitted network model. This is followed by C_2 from the AI Configuration Anxiety dimension (“I find humanoid AI technologies (e.g., humanoid robots) intimidating,”* R*^2^ = 0.917), L_2 from the Learning Anxiety dimension (“Learning to use AI technologies makes me anxious,”* R*^2^ = 0.907), and C_3 from the AI Configuration Anxiety dimension (“I don't know why, but humanoid AI technologies (e.g., humanoid robots) scare me,”* R*^2^ = 0.896).

### 3.4 Accuracy and stability analysis of the AI anxiety network

#### 3.4.1 Edge weight accuracy

**Figure 3:**
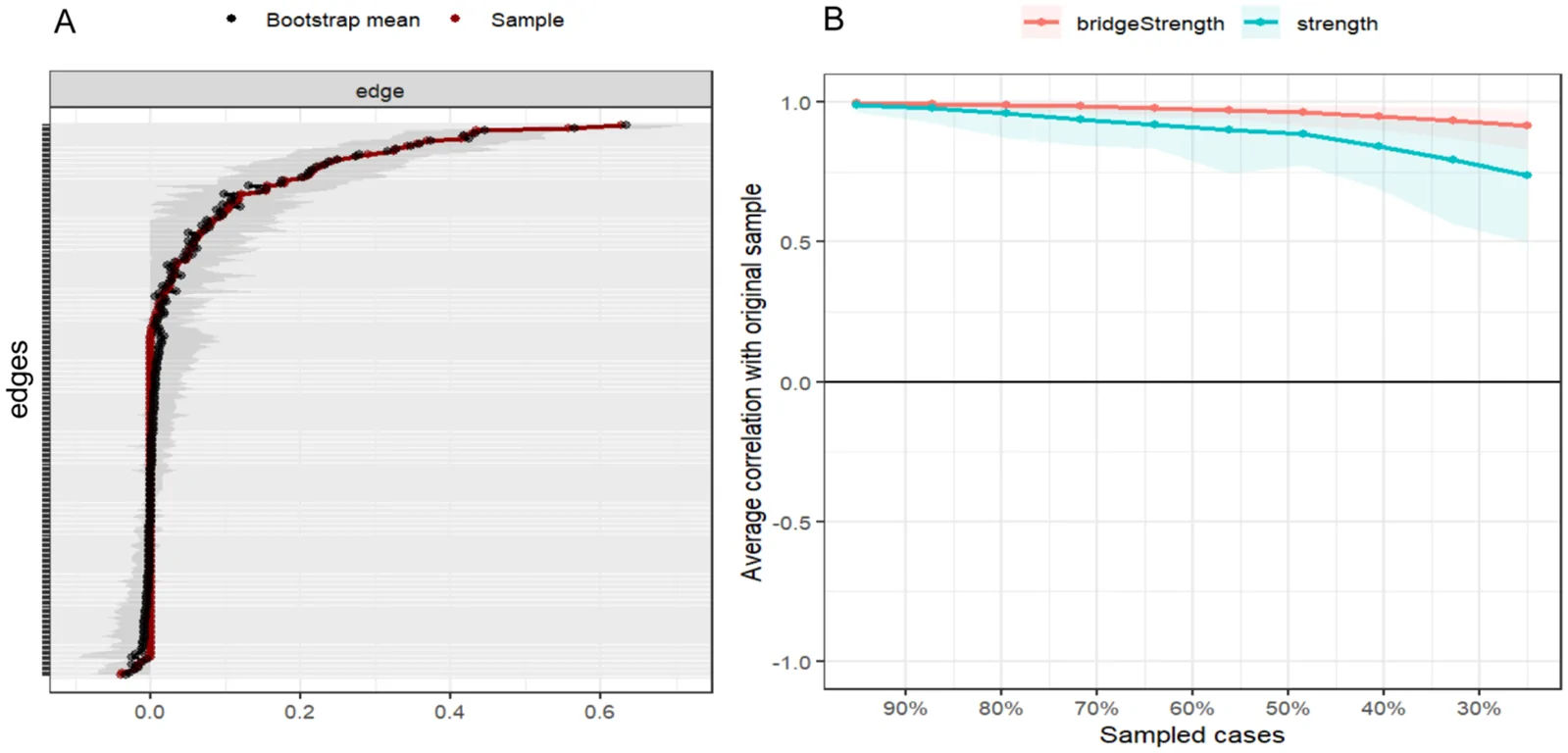
Edge-weight accuracy and centrality stability of the AIAS network. **(A)** Edge-weight accuracy: this panel displays the 95% nonparametric bootstrap confidence intervals (CIs) for the estimated edge weights based on 1,000 bootstrap resamples. The red line represents the sample edge weights, the black line represents the bootstrap mean edge weights, and the gray shaded areas represent the 95% CIs. Narrower confidence intervals indicate greater precision and lower uncertainty in the estimated edge weights. **(B)** Centrality stability (case-dropping bootstrap): this panel illustrates the stability of node strength (blue line) and bridge strength (red line) using the case-dropping bootstrap method. The x-axis represents the percentage of cases retained in the subsampled datasets, and the y-axis represents the average correlation between the centrality indices estimated from the original sample and those estimated from the subsamples. The shaded areas denote 95% CIs. Stability is quantified using the correlation stability coefficient (CS coefficient); a value above 0.50 is generally considered indicative of acceptable stability of the relative centrality rankings. This stability does not establish the causal or clinical importance of individual nodes.

Figure 3A presents the accuracy analysis of the AI anxiety network's edge weights, evaluated using 95% non-parametric confidence intervals (CIs) calculated from 1,000 bootstrap samples (28). The results show that most edge confidence intervals are relatively narrow, indicating relatively precise edge-weight estimates. Specifically, edges with larger weights (>0.2) tended to have narrower confidence intervals, suggesting that these key associations are estimated with high reliability. However, some edge weights (particularly those close to zero) exhibit wider confidence intervals, indicating greater estimation uncertainty for these edges. This suggests caution when interpreting weaker associations in the network, especially secondary associations in nurses' AI anxiety, to avoid overinterpretation and ensure the robustness of conclusions.

#### 3.4.2 Centrality stability

Figure 3B presents the stability analysis of strength centrality and bridge strength, assessed using the correlation stability coefficient (CS coefficient). The results indicate that the CS coefficient for strength centrality is 0.594, and for bridge strength, it is 0.750, both exceeding the preferred threshold of 0.5 (17). These coefficients indicate acceptable stability of the estimated strength and bridge-strength rankings under case-dropping procedures. Combined with the results shown in Figure 2, they support the reproducibility of the relative network positions of nodes such as C_2 and S_1 within this sample, but do not establish their causal or clinical importance.

### 3.5 Within-network difference testing

**Figure 4:**
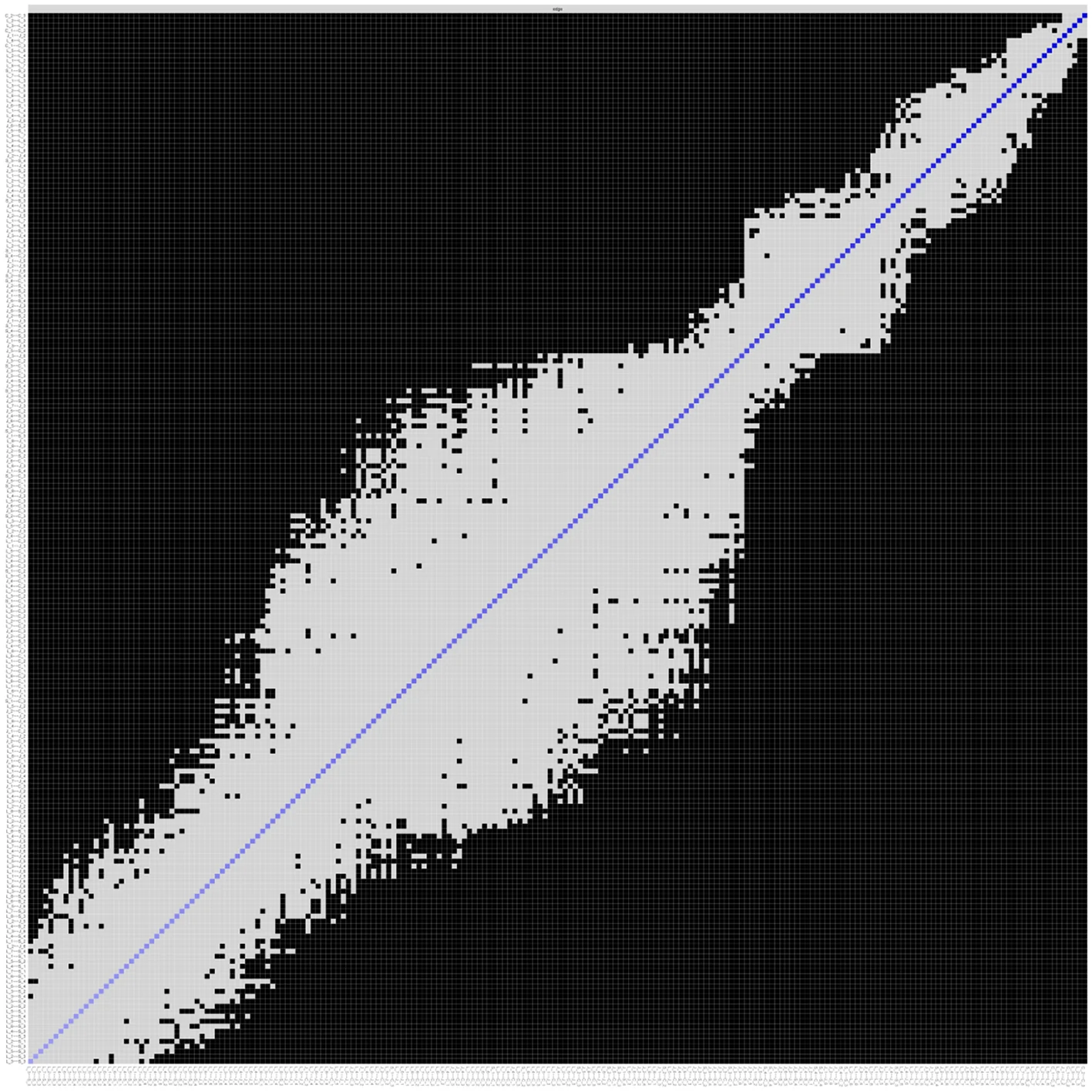
Bootstrapped difference test of edge weights in the AIAS network. This matrix presents the results of the bootstrap difference test, based on 1,000 bootstrap resamples, comparing all pairs of estimated edge weights within the AIAS network. The x-axis and y-axis represent individual edges. Black cells indicate statistically significant differences between two edge weights, whereas gray cells indicate non-significant differences. The values along the diagonal represent the estimated edge weights. A larger number of black cells indicates that a greater number of edge-weight pairs were statistically distinguishable from one another. This difference test does not by itself establish the overall stability or reliability of the network.

**Figure 5:**
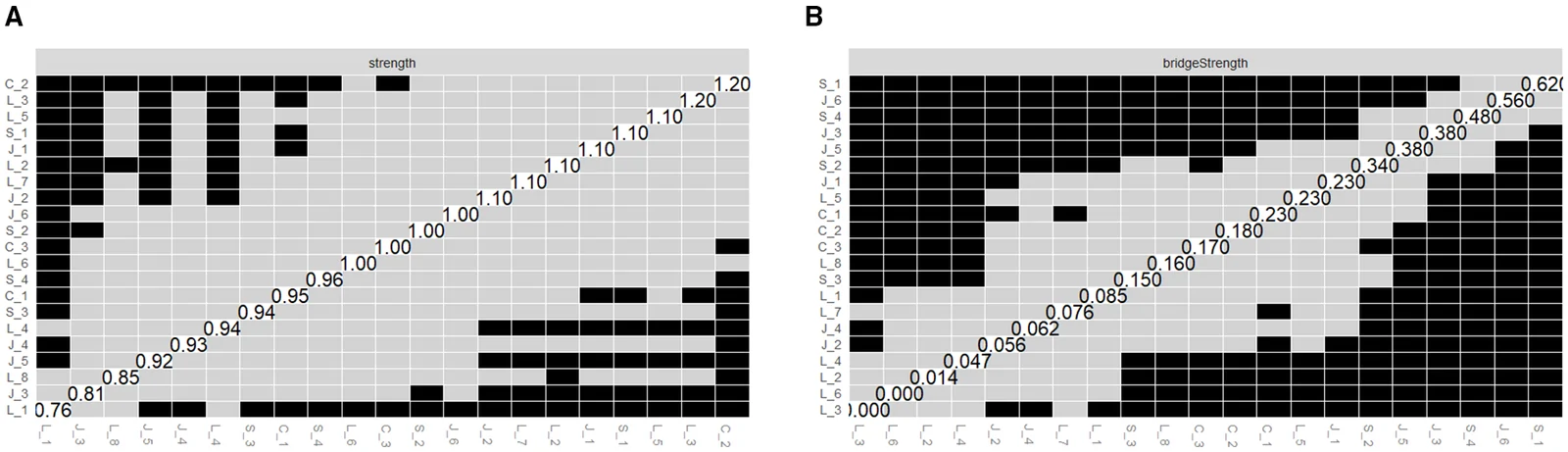
**(A)** Bootstrapped difference test for node strength. This matrix presents pairwise statistical comparisons of node strength based on 1,000 bootstrap resamples. The x-axis and y-axis represent individual nodes in the AIAS network. Black cells indicate statistically significant differences at the 0.05 level, whereas gray cells indicate non-significant differences. The values along the diagonal represent the estimated node-strength values. The relatively large number of significant comparisons involving C_2 and L_3 indicates that their estimated strength values differed significantly from those of several other nodes. These differences reflect relative statistical connectivity within the estimated network and do not establish causal influence or intervention priority. **(B)** Bootstrapped difference test for node bridge strength. This matrix presents pairwise statistical comparisons of node bridge strength based on 1,000 bootstrap resamples. As in panel A, black cells indicate statistically significant differences at the 0.05 level, whereas gray cells indicate non-significant differences. The values along the diagonal represent the estimated bridge-strength values for each node. The relatively large number of significant comparisons involving S_1 and J_6 indicates that their estimated bridge-strength values differed significantly from those of several other nodes. These findings indicate relatively high cross-dimensional statistical connectivity within the estimated network but do not establish that these nodes transmit anxiety between dimensions.

Figures 4, 5 present the results of bootstrap difference tests comparing edge weights, node strength centrality, and bridge strength centrality within the AI anxiety network.

#### 3.5.1 Edge weight difference testing

Figure 4 displays the results of the bootstrap difference test for edge weights in the AI anxiety network. In the matrix diagram, black squares indicate statistically significant differences between pairs, while gray squares indicate non-significant differences, with the diagonal line representing the specific values of the edges. A larger number of black squares indicates that more pairs of edge weights differ significantly, suggesting greater distinguishability among the estimated edges. In this study, several key edges exhibited significant differences, particularly those related to L_3 from the Learning Anxiety dimension (“Learning to use specific functions of AI technologies makes me anxious”) and C_2 from the AI Configuration Anxiety dimension (“I find humanoid AI technologies (e.g., humanoid robots) intimidating”).

#### 3.5.2 Node strength centrality difference testing

Figure 5A presents the results of the bootstrap difference test for node strength centrality. The diagonal of the matrix shows the strength centrality values for each node, with gray boxes indicating non-significant differences and black boxes indicating significant differences. The results reveal that C_2 from the AI Configuration Anxiety dimension (Strength = 1.488, Figure 2A) and L_3 from the Learning Anxiety dimension (Strength = 1.350, Figure 2A) exhibit significant differences compared to most other nodes, as indicated by a higher number of black boxes in their corresponding rows. This indicates that the estimated strength values of C_2 and L_3 were higher than those of many other nodes in the network.

#### 3.5.3 Node bridge strength centrality difference testing

Figure 5B presents the results of the bootstrap difference test for node bridge strength, with a matrix structure similar to that of Figure 5A. The results show that S_1 from the Sociotechnical Blindness dimension (“I am afraid that AI technologies may be misused,” Bridge Strength = 2.215, Figure 2B) and J_6 from the Job Replacement Anxiety dimension (“I am afraid that AI technologies will replace someone's job,” Bridge Strength = 1.899, Figure 2B) exhibit significant differences compared to most other nodes, as evidenced by the pre-dominance of black boxes in their corresponding rows. This indicates that S_1 and J_6 showed relatively high bridge-strength values, indicating strong cross-dimensional connectivity within the network (e.g., Learning Anxiety and Job Replacement Anxiety), highlighting the statistical prominence of AI misuse and job-replacement concerns within the estimated network structure.

The difference tests showed that the strength or bridge-strength values of C_2, L_3, S_1, and J_6 were significantly higher than those of several other nodes, indicating their relatively high statistical prominence within the estimated network. These nodes represent nurses' concerns regarding humanoid AI technologies, learning AI functions, AI misuse, and job replacement, respectively. These findings may generate hypotheses for subsequent intervention studies, such as prospectively evaluating AI training programs that address these concerns. They do not establish that the identified nodes are causal drivers or optimal intervention targets.

### 3.6 Association between AI anxiety and willingness to receive AI-related training

**Figure 6:**
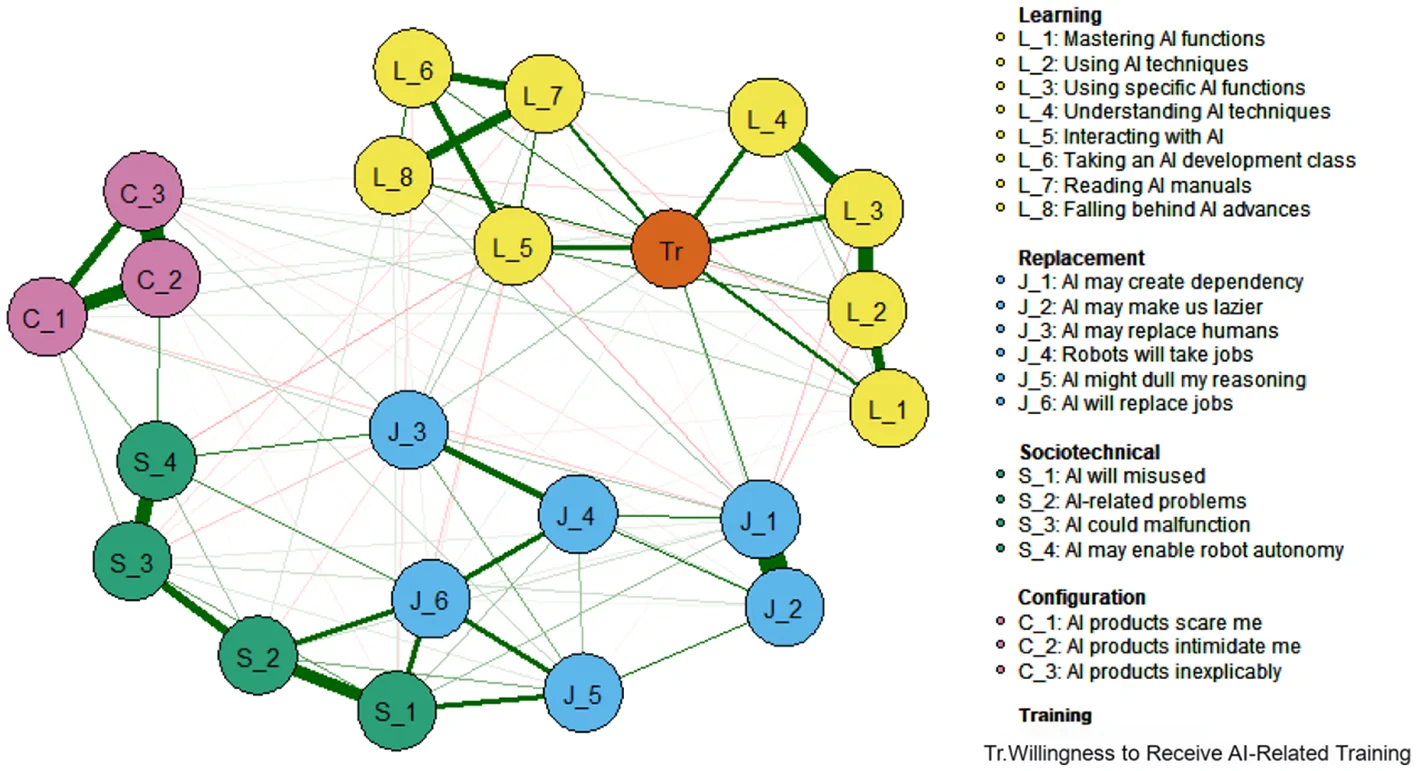
Network associations between AI anxiety items and willingness to receive ai-related training. This figure illustrates the integrated network analysis incorporating the 21 AIAS items and the specific node representing willingness to receive AI-related training (Tr). Nodes represent individual anxiety items and the global rating of willingness to receive AI-related training, color-coded by dimension: Learning Anxiety (yellow, L1-L8), Job Replacement Anxiety (blue, J1-J6), Sociotechnical Blindness (green, S1-S4), AI Configuration Anxiety (pink/purple, C1-C3), and Willingness to Receive AI-Related Training (orange, Tr). Edges (lines) represent the regularized partial correlation coefficients calculated via the LASSO method. The thickness of an edge reflects the strength of the association, while the color indicates the direction (green for positive, red for negative). Learning-anxiety items L_5, L_4, and L_3 showed the strongest positive conditional associations with the global rating of willingness to receive AI-related training. These associations do not establish directionality or indicate specific multidimensional training needs.

Figure 6 illustrates the network associations between the 21 AIAS items and willingness to receive AI-related training (Tr). The results reveal that items from the Learning Anxiety dimension exhibit the strongest associations with willingness to receive AI-related training. Specifically, L_5 (“Learning to interact with AI technologies makes me anxious”) shows the highest edge weight with willingness to receive AI-related training (Edge Weight = 0.24), indicating that nurses' anxiety about interacting with AI technologies is closely related to their willingness to receive AI-related training. Following this, L_4 (“Learning how an AI technology works makes me anxious,” Edge Weight = 0.23) and L_3 (“Learning to use specific functions of AI technologies makes me anxious,” Edge Weight = 0.22) also demonstrate strong associations. These associations reflect the single global rating of training willingness and do not indicate preferences for particular training content, formats, competency levels, or delivery modalities.

## 4 Discussion

This study used network analysis to characterize the item-level structure of AI anxiety among Chinese maternal and child health nurses and its association with a global rating of willingness to receive AI-related training. The findings identify statistically prominent anxiety items and generate hypotheses for prospective research and organizational support.

### 4.1 Interpretation of the AI anxiety network structure and statistically central items

The network analysis results indicate that within the AI anxiety network, C_2 within the AI Configuration Anxiety dimension (“I find humanoid AI technologies (e.g., humanoid robots) intimidating,” Strength = 1.488) and L_3 within the Learning Anxiety dimension (“Learning to use specific functions of AI technologies makes me anxious,” Strength = 1.350) exhibit the highest strength centrality. In statistical network terms, this suggests that humanoid AI fear and learning anxiety occupied relatively central positions within the AI anxiety profile of maternal and child health nurses. One possible contextual interpretation is that, in specialized settings where human-to-human empathy and maternal-infant bonding are highly valued, some nurses may perceive humanoid AI as potentially challenging the humanistic core of nursing care. This interpretation was not directly tested in the present study.

This finding aligns with existing research. Nyholm et al. (29) observed that healthcare professionals' fear of humanoid AI often stems from psychological discomfort triggered by anthropomorphic features, which may be intertwined with other anxiety dimensions, such as job replacement anxiety. These findings are also consistent with a previous self-efficacy network study in which “anthropomorphic interaction” (e.g., AI tone matching humans) showed relatively high centrality, indicating that human-like attributes of AI were statistically prominent within that estimated network (11). Similarly, Cho et al. (30) found that learning anxiety was prevalent among nursing students, particularly when they faced complex technological functions, and that greater technological unfamiliarity was associated with higher anxiety levels. This is consistent with the high strength centrality of L_3 observed in the present study.

Furthermore, bridge strength analysis revealed that S_1 from the Sociotechnical Blindness dimension and J_6 from the Job Replacement Anxiety dimension showed the highest bridge-strength values. Within the estimated network, S_1 displayed relatively strong cross-dimensional connections involving concerns about AI misuse and learning-related anxiety, whereas J_6 showed cross-dimensional connectivity involving job-replacement and AI-configuration concerns. These findings describe conditional association patterns and do not establish that one concern causes, transmits, or amplifies another (26).

The identified central and bridge nodes should be interpreted as statistically prominent, hypothesis-generating concerns rather than causal drivers or validated intervention targets. Centrality describes a node's position in an estimated cross-sectional network; whether modifying a node changes other components requires longitudinal or interventional evidence (17, 28).

### 4.2 Theoretical value of network predictability and stability

Node predictability ranged from 69.3 to 92.1%. L_3 (“Learning to use specific functions of AI technologies makes me anxious,”* R*^2^ = 0.921) and C_2 (“I find humanoid AI technologies [e.g., humanoid robots] intimidating,”* R*^2^ = 0.917) showed the highest predictability, indicating that much of their variance was statistically accounted for by neighboring nodes in the fitted network. This reflects strong multivariate connectivity around L_3 and C_2 but does not identify the contribution of any individual neighboring item.

The CS coefficients for strength centrality and bridge strength were 0.594 and 0.750, respectively, both exceeding the preferred threshold of 0.50 (17). These results support the stability of the relative node rankings within this sample.

Compared with traditional variable-centered approaches, network analysis provides an item-level structural perspective by characterizing conditional associations among AI anxiety items, without requiring these associations to be interpreted as temporal or causal interactions (16). This is particularly important for maternal and child health nurses, as they have received limited attention in AI anxiety research. Although the pre-dominance of female participants (97.1%) may reflect the sex composition of the maternal and child health nursing workforce in many settings, the small number of male participants precluded sex-based comparisons and limits the applicability of the estimated network structure to male nurses and more sex-balanced nursing populations. In addition, 43.6% of the participants had never received AI-related training. The mean scores for difficulty in learning specific AI functions (L_3, mean 4.0) and concerns regarding technological dependency (J_1, mean 4.65) indicate that these issues were salient within the overall sample. These items were embedded in an interconnected statistical network, indicating that they were conditionally associated rather than statistically isolated within the fitted model.

By mapping item-level conditional associations, this study extends AI-anxiety research in maternal and child health nursing and generates testable hypotheses. For example, modular training for learning-related concerns or ethics education for misuse concerns may be evaluated prospectively, but their effectiveness cannot be inferred from the present network.

### 4.3 The relationship between AI anxiety and training willingness and its organizational support implications

The learning-anxiety items L_5, L_4, and L_3 showed the strongest associations with willingness to receive AI-related training (edge weights = 0.24, 0.23, and 0.22, respectively). These data indicate that greater learning-related anxiety co-occurred with greater global training willingness, but they do not establish directionality or specify educational needs. Because willingness was assessed with a single item, the study cannot identify preferred content, learning modality, competency level, ethical topics, or implementation support. Othman et al. (7) reported lower AI-related anxiety after training, supporting prospective evaluation rather than immediate implementation.

In the present sample, 43.6% of nurses had no previous exposure to AI-related training and 13.7% had received specialized training. This descriptive pattern coexisted with moderate learning-anxiety scores, but the study did not compare anxiety by prior training exposure or professional title; therefore, subgroup-specific training priorities cannot be inferred.

Accordingly, targeted AI training should be regarded as a literature-informed organizational option for prospective evaluation, not as an intervention tested by this study or a participant-derived preference. Institutions should first conduct multidimensional needs assessments and then evaluate whether phased instruction, simulation, ethics education, or multilevel support improves competence, adaptation, or other relevant outcomes.

### 4.4 Practical implications of training strategies and organizational support

Based on the observed associations, the following strategies are presented only as literature-informed candidates for prospective evaluation. They were neither directly tested nor elicited as participant preferences; therefore, their content and delivery should be determined through multidimensional needs assessment and stakeholder co-design.

(1) Step-by-step training addressing learning-related concerns
  Phased, competency-oriented instruction may be evaluated for nurses with limited AI exposure, progressing from foundational digital operations to AI-assisted clinical applications (31). Its effects on competence, anxiety, and professional outcomes require interventional testing.
(2) Simulation-based interactive courses addressing humanoid-AI fear
  Simulation-based familiarization with humanoid AI may be evaluated because humanoid-AI fear was statistically prominent and simulation has supported communication-skills training in nursing education (32). The present findings do not establish this concern as a causal target or show that simulation will reduce anxiety.
(3) Ethics and safety education addressing concerns about job replacement and AI misuse
  Ethics and safety education may address literature-described concerns about AI misuse, algorithmic bias, privacy, and job displacement (10, 33). Whether such education changes anxiety or acceptance among maternal and child health nurses requires prospective evaluation.
(4) Multilevel training system to meet diverse career needs
  Because AI acceptance may vary by professional level (9), multilevel programs could be evaluated; however, the present study did not test professional-title differences. Any tiered design should therefore be guided by formal needs assessment rather than inferred from the network.
(5) Continuous support mechanism to reinforce training effects
  Continuing education, consultation, and feedback mechanisms may support AI integration (34), but their effectiveness in this population was not examined. Institutions with different AI infrastructure and training resources should adapt and evaluate these approaches locally.
  These strategies are most directly relevant to institutions resembling the study sample, which was dominated by teaching hospitals (71.8%) and tertiary Grade A hospitals (57.9%). Their transferability to non-teaching, lower-level, resource-limited institutions and broader nursing populations remains uncertain.
  Before implementation, nursing leaders should conduct multidimensional needs assessments, involve nurses in program co-design, and prospectively evaluate outcomes such as competence, anxiety, workload, and professional wellbeing.

## 5 Limitations and future research directions

Although this study characterized the network structure of AI anxiety and its association with willingness to receive AI-related training, several limitations should be considered. First, the cross-sectional design and undirected network preclude temporal or causal inference. The identified central and bridge nodes cannot be considered intervention targets, and the proposed organizational strategies require longitudinal or experimental evaluation.

AI anxiety and training willingness were self-reported in the same survey at one time point, creating potential common method bias despite anonymous data collection and standardized instructions. Future studies should use temporally separated assessments, multiple data sources, or objective indicators of training participation.

Second, convenience sampling and the highly specific study population limit representativeness. Participants were maternal and child health nurses in mainland China; 97.1% were female, 92.1% were Han, 71.8% worked in teaching hospitals, and 57.9% worked in tertiary Grade A hospitals. Because AI infrastructure, training access, and organizational support may differ across settings, the findings may not generalize to male nurses, ethnic minority groups, other nursing specialties or countries, or non-teaching, lower-level, and resource-limited institutions.

Eligibility was also restricted to nurses engaged in routine clinical and teaching duties, which may have underrepresented those in full-time academic, research, or external training roles. Future studies should use probability-based, stratified, or quota sampling and examine whether the network structure differs across hospital level, teaching status, region, sex, ethnicity, and professional role.

Third, willingness to receive AI-related training was measured using one global item. This captured overall willingness but not multidimensional educational needs, preferences, competency priorities, or barriers. Accordingly, the strategies discussed are literature-informed hypotheses rather than participant-derived requirements. Future studies should use multidimensional measures and qualitative or co-design methods.

Additionally, although the AIAS covers multiple dimensions of anxiety, it may not fully capture the specific sources of anxiety faced by maternal and child health nurses, such as unique pressures arising from AI-mediated communication with pregnant women and their families. Future research could develop specialized scales for maternal and child health contexts to improve measurement precision and relevance.

The reliance on quantitative data also limited exploration of nurses' subjective experiences and contextual mechanisms. Mixed-methods studies could examine why learning anxiety and humanoid-AI fear are salient and inform context-specific intervention design.

This study did not assess nursing performance, care quality, job satisfaction, professional wellbeing, or retention, nor potentially influential factors such as resource availability, salary, workload, staffing, and leadership support. Future longitudinal and multivariable studies should measure these outcomes and covariates when evaluating AI-related training and organizational support.

Finally, although expert review, content-validity assessment, pilot testing, and internal-consistency analysis supported the contextual applicability of the AIAS, the present study did not conduct a full psychometric revalidation, such as confirmatory factor analysis or measurement-invariance testing, in maternal and child healthcare nurses. Future studies should further examine its factorial validity and cross-context measurement properties in this population.

Future longitudinal, mixed-methods, psychometric, and intervention studies should test the temporal structure of AI anxiety and evaluate context-sensitive training strategies across more diverse nursing populations.

## Data Availability

The original contributions presented in the study are included in the article/supplementary material, further inquiries can be directed to the corresponding author.
